# Novel insights into m^6^A modification of coding and non-coding RNAs in tumor biology: From molecular mechanisms to therapeutic significance

**DOI:** 10.7150/ijbs.73093

**Published:** 2022-07-04

**Authors:** Jinlin Jia, Suwen Wu, Zimo Jia, Chang Wang, Chenxi Ju, Jinxiu Sheng, Fucheng He, Mingxia Zhou, Jing He

**Affiliations:** 1 Department of Medical Laboratory, The First Affiliated Hospital of Zhengzhou University, Zhengzhou 450052, China; 2 Obstetrics and Gynecology Hospital, Fudan University, Shanghai 200011, China; 3 Department of Biochemistry and Molecular Biology, Hebei Medical University, Shijiazhuang 050017, China; 4 Department of Gastroenterology, The First Affiliated Hospital of Zhengzhou University, Zhengzhou 450052, China; 5 Department of Breast Surgery, The First Affiliated Hospital of Zhengzhou University, Zhengzhou 450052, China

**Keywords:** m^6^A, non-coding RNA, tumor microenvironment, therapeutic target

## Abstract

Accumulating evidence has revealed that m^6^A modification, the predominant RNA modification in eukaryotes, adds a novel layer of regulation to the gene expression. Dynamic and reversible m^6^A modification implements sophisticated and crucial functions in RNA metabolism, including generation, splicing, stability, and translation in messenger RNAs (mRNAs) and non-coding RNAs (ncRNAs). Furthermore, m^6^A modification plays a determining role in producing various m^6^A-labeling RNA outcomes, thereby affecting several functional processes, including tumorigenesis and progression. Herein, we highlighted current advances in m^6^A modification and the regulatory mechanisms underlying mRNAs and ncRNAs in distinct cancer stages. Meanwhile, we also focused on the therapeutic significance of m^6^A regulators in clinical cancer treatment.

## Introduction

Extensively investigations have revealed that m^6^A RNA modification is a reversible and dynamic complex biological process, among which a series of proteins are recruited to the bound regions to execute their functions 1. m^6^A modification typically accounts for 0.1%-0.4% of total cellular RNA adenosines. Previous studies have validated that m^6^A modification sites are mainly located close to stop codons, or the 5'- and 3'-untranslated regions (UTRs) 2, 3. The m^6^A modification is frequently observed in the long internal exon in the vast genome of mRNA, which indicates that it is intrinsically connected with mRNA functions 1, 4-6. Additionally, m^6^A modification sites also exist in the unique motifs of diverse ncRNAs, including miRNAs, long non-coding RNAs, and circRNAs 7, 8. The m^6^A modification complements are associated with dysregulation of RNAs, including mRNA and ncRNAs, responsible for cancer's hallmarks, such as sustaining proliferative signaling, resisting cell death, evading growth suppressors, inducing angiogenesis, avoiding immune destruction, etc. 9-14. Therefore, we can conclude that there are intricate potential interaction networks between m^6^A and mRNA or ncRNA, by which m^6^A and mRNA or ncRNA co-modulate target mRNAs via counteraction, orchestration or both.

Although still in inception, outstanding efforts have been devoted to scrutinizing the complicated crosstalk between m^6^A modification and RNAs. In this review, we summarized the latest proceedings concerned with the interaction between m^6^A modification and RNAs by describing the biological effect and underlying regulatory mechanisms of m^6^A modified RNAs and the influences of m^6^A-RNAs on human malignancies. Finally, the prospect of m^6^A in cancer diagnosis and therapy was heavily discussed.

## 1. The effect and regulation of m^6^A in mRNA

### 1.1 m^6^A writers

The biological function of m^6^A modification is dedicatedly governed by m^6^A methyltransferase (also called m^6^A writers), m^6^A demethylase (also called m^6^A erasers), and m^6^A binding protein (also called m^6^A readers) 15. The first widespread dynamic mRNA modification, in conjunction with other RNA chemical modifications, has coined the epitranscriptomics field and m^6^A has become the spotlight in this emerging territory. Nowadays, compelling evidence has validated a series of m^6^A molecular compositions.

M^6^A methyltransferase-like3 (METTL3) and methyltransferase-like 14 (METTL4) form the multicomponent m^6^A methyltransferase complex (MTC), which executes the m^6^A modification function 16. The former binds to the methyl donor S-adenosyl methionine (SAM) and catalyzes methyl group transfer depending on its highly active methyltransferase domain. Meanwhile the latter is essential for m^6^A deposition by forming a heterodimer with METTL3 and substrate recognition, contributing to the methylase activity 17. At the same time, auxiliary protein participates in the MTC assembly to ensure the stability of the METTL3-METTL14 complex. Wilms tumor 1-associated protein (WTAP) was the first identified auxiliary protein to reciprocate with the METTL3-METTL14 heterodimer 4. Moreover, MTC consists of key regulatory factors, including via-like m^6^A methyltransferase associated (VIRMA, also known as KIAA1429), zinc finger CCCH domain-containing protein 13 (ZC3H13), and RNA-binding motif protein 15 (RAB15/15B), which play critical roles in stabilizing core complex 18. However, the precise mechanisms by which these adaptor proteins control MTC formation remain to be elucidated.

Distinctive from what has been observed within the m^6^A chemical mark catalyzed by MTC, recent research has pinpointed that methyltransferases like 5 (METTL5), methyltransferases like 16 (METTL16), and zinc finger CCHC-type containing 4 (ZCCHC4) execute m^6^A methyltransferase function in an MTC-independent manner 19-21. For example, the methyltransferase activity of ZCCHC4 is independently mediated by an integrated 28S RNA-binding surface, comprising of three domains 19. Except for the typical ZCCHC4 methyltransferase domains, there are two specific structures involved in the RNA-binding site: The N-terminal specific zinc finger domain and the C-terminal CCHC domain 19. We can deduce that this ubiquitous m^6^A chemical remark harbors multiple complicated mechanisms, in contrast to except for what has been observed in the METTL family.

### 1.2 m^6^A erasers

The m^6^A modification has been shown to be a reversible and dynamic biological process ever since the functional identification of the fat mass and obesity-associated protein (FTO), the first reported human m^6^A erasers to be described 22. ALKB homolog 5 (ALKBH5) has been identified as the second m^6^A eraser, which is also capable of demethylating m^6^A modification 23. The demethylation characters embedded in FTO and ALKBH5 can remove the m^6^A remark labeled in mRNAs and ncRNAs. They promote demethylation reactions and ebb m^6^A modification level in cellular mRNA, which is essential for dynamic balance in the m^6^A regulation network. In contrast to being exclusively localized in cell nuclei or nucleoplasm, FTO is partly distributed in a spotty form throughout the nucleoplasm and partially colocalized within the nuclear splicing speckles 22. These splicing speckles such as RNA polymerase II phosphorylated at Ser2 (Pol II-S2P) functions in the spliceosome assembly 22. Moreover, FTO is partially located in nuclei and could be recruited to the assembly spliceosome center by communicating with its nuclear speckles partner, finally facilitating mRNA processing 22. Similar to FTO, ALKBH5 has been identified as a factor in mRNA export, connecting RNA metabolism to nuclear speckle. Interestingly, ALKBH5 has been certified as a key determinant in spermatogenesis and the p53 signal pathway network 23.

Maintaining the stability of m^6^A modification levels in endogenous RNAs is indispensable for diverse cellular activities under physiological situations. The equilibrium between m^6^A modification deposition and removal serves a functional purpose in the dynamic interactive network. As a result, mutation or deregulation of the m^6^A composer is intrinsically linked to human cancers. Overpowering research has validated the oncogenic and tumor-suppressing effects of these writers and erasers in cancer. In addition, the non-homeostasis caused by writers alone, erasers alone, or both could result in the aberrant m^6^A modification in the critical and functional RNA transcripts which have a significant role in tumorigenesis and progression.

### 1.3 m^6^A readers

Besides writers and erasers, the m^6^A regulation system also contains one type of unique RNA-binding protein, called m^6^A readers. There have been distinct complex regulatory mechanisms modulating gene expression during transcription, post-transcription, and following gene expression procedures such as translation. The m^6^A modification can occur in infant RNA transcripts in the nucleus during transcription. The m^6^A methylation installed in RNA also impacts the subsequent gene expression mediated by diverse mechanisms, such as altering RNA local structure or recruiting selective m^6^A readers to the bound m^6^A remarked RNA 16.

The mRNA alternative splicing (AS) event occurs in nucleus speckles, where m^6^A writers and erasers to accumulate 4, 24. Most recent studies have revealed that m^6^A writers and erasers directly or indirectly interact with nearby splicing speckles that function as critical mRNA splicing factors in AS or pre-mRNA processing 4, 24. This cellular location pattern demonstrates that m^6^A modification is closely linked to mRNA splicing. RNA transcripts distributed in the nucleus and nucleoplasm possess fundamentally distinct fates. Regarding the nucleus, nascent transcripts tend to produce mature signals devoid of introns. In the cytoplasm, mRNA can be stored in specific functional carriages, including messenger ribonucleoprotein (mRNP) foci, actively translating, stabilizing, or degrading. Active protein complexes involving gene expression vary from nuclei such as mRNA AS and export to cytoplasm such as translation and decay. Based on this, there has been one distinct m^6^A reader mechanism notion: m^6^A readers could be categorized as either nuclear readers, such as heterogeneous nuclear ribonucleoproteins A2/B1 (HNRNPA2B1) and YTH domain-containing protein 1 (YTHDC1) or cytoplasmic readers such as YTH domain family proteins (YTHDF1-3), which bind specific to a m^6^A pocket within the nucleus or cytoplasm, respectively 25.

#### Nuclear m^6^A readers

The RNA binding protein YTHDC1 is located in YT bodies, which are spatially in proximity with nuclear speckles recruiting AS factors, implying that YTHDC1 participates in mRNA splicing 26. SR family proteins have been demonstrated to function as a crucial splicing factor by activating exon inclusion or exon skipping 27. The splicing mechanism between SRSF3 and SRSF10 is distinct from each other. SRSF3 facilitates exon inclusion in trans-acting splicing. Interestingly, the C-terminal RS domain of SRSF3 and SRSF10 competitively binds with the N-terminal YTHDC1 competitively 28. In the nucleus, YTHDC1 recruits SRSF3 to form a protein-protein interaction complex and brings SRSF3 to the mRNA-binding element by binding to the m^6^A-bearing mRNA, changing the splicing pattern 28.

Meanwhile, the interaction between YTHDC1 and SRSF10 impedes exon skipping of SRSF10 in the mRNA-binding element 28. In addition, research has reported that YTHDC1 shortens the residence time of m^6^A modification mRNA in the nucleus, thereby reducing the subcellular abundance of target transcripts in the nucleus and conversely promoting the accumulation of transcripts in the cytoplasm 29. YTHDC1 regulates the distribution of cellular transcripts and facilities m^6^A-mRNA transport from the nucleus to the cytoplasm by interacting with SRSF3 29. Indeed, nucleus reader proteins binding m^6^A possess other protein-protein interaction domains, which communicate with the bound downstream responding elements to specify the key activities.

Besides engaging in AS, YTHDC1 has been validated to fine-tune transcription and gene expression by different mechanisms. YTHDC1 binding with m6A-modified transcripts recruits H3K9me2 demethylase, which diminishes transcriptional repression in the chromatin domains of m^6^A-mRNA 30. Moreover, it tunes chromatin accessibility and transcription by facilitating chromosome-associated regulatory RNAs (carRNAs) degradation, mediated by nuclear exosome targeting-mediated degradation 31. Also, YTHDC1 governs transcription and gene expression by interacting with enhancer RNAs (eRNAs), or ncRNAs 32. YTHDC1 increases enhancer activity by perceiving m^6^A modified eRNA and recruits transcriptional activators BRD4, which is implicated in phase-separated transcriptional condensates 33. ncRNAs will be discussed in the following section. Herein, we can conclude that the complex and dynamic roles of YTHDC1 in the nucleus may be associated with RNA types such as typical mRNAs, car RNAs, and lncRNAs.

#### Cytoplasm reader

This working mechanism gets along with cytoplasm reader proteins well. According to translational activities, mRNA present in the cytoplasm is divided into three function pools: non-ribosome mRNA-protein particles (the lowest translational viability, mRNA decay sites), translatable mRNA pool mRNPs related to translation effectors (middle translational viability), and actively translating polysome (high translation viability) 34. The C-terminal domain of YTHDF2 has been confirmed to recognize the core motif of G(m^6^A)C in the bound mRNA. This interaction transfers target mRNA from translatable pool to processing bodies or other mRNA degrading compartments mediated by the N-terminal domain 35. However, YTHDF1 preferentially delivers m^6^A-bearing mRNAs into the translation pool and increases the translation initiation efficiency of m^6^A remarked mRNA loading with ribosomes by interacting with ribosomes and eIF3 in an RNA-independent manner 36. Simultaneously, YTHDF1 collaborates with trans-acting regulators such as insulin-like growth factor 2 mRNA-binding protein1 (IGF2BP1), YBX1, G3BP1 and poly(A) binding protein cytoplasmic 2 (PCBP2) in an RNA-dependent pattern. Translation and decay are two adverse gene expression outputs. The translation-promotion influence of YTHDF1 and the decay-promotion effect of YTHDF2 on mRNA fate are two opposite mechanisms. Indeed, YTHDF2 and YTHDF1 share abundant target transcripts besides their specific target transcripts 36. The latter combines target m^6^A sites located in these common mRNAs earlier than the former. YTHDF2 and YTHDF1 corroborate to modulate the translation efficiency of dynamic transcripts 36. Recent research has suggested that YTHDF3 facilitates protein synthesis by combining with YTHDF1 and promotes mRNA decay by interacting with YTHDF2 37. We can conclude from all these studies that three YTHDF proteins contribute to cytoplasmic m^6^A mRNA metabolism in the cytoplasm in an integrated and cooperative mechanism.

Growing evidence has demonstrated that emerging m^6^A readers such as IGF2BPs (IGF2BP1/2/3) and HNRNPA2B1 recognize m^6^A sites and modulate mRNA life cycle 25, 38. As is well established, IGF2BP elevates the stability of m^6^A modified mRNA and promotes translation by its K homology domains (KH domain)- containing m^6^A binding site 38. METTL3 has been identified as an m^6^A reader as studies have confirmed that it promotes mRNA translation in an m^6^A-independent manner 39. Furthermore, it interacts with eIF3 subunit h (eIF3h) to form the METTL3-eIF3h loop, enhancing protein synthesis by ribosome cycling in a manner analogous to eIF4G-PABPC1 (poly(A)-binding protein) mediated mRNA looping 40. Notably, mRNA metabolism events in the nuclear and cytoplasm do not occur independently. Diverse mechanisms have allowed frequent information communications and crosstalk between the nucleus and cytoplasm. We speculate that m^6^A modification plays a key role in coordinating RNA fundamental biological processes between the nucleus and cytoplasm.

In addition to participating in mRNA metabolism, m^6^A alters local secondary RNA structure. It regulates the accessibility of RNA binding motifs (RBM), suggesting that m^6^A affects the RNA-protein interaction to produce a widespread and far-ranging influence on biological processes 41, 42. This working mechanism is also termed the m^6^A switch. For example, m^6^A modification in mRNA increases the spatial affinity of its surrounding protein binding elements to the RBM of HNRNP C, which is best known to be associated with pre-mRNA processing in AS 43. The accessibility of mRNA to bind Arg-Gly-Gly repeats included in the low-complexity domain of HNRNPG is enhanced once upon the target mRNA is exposed to m^6^A, changing the AS pattern of the m^6^A modified mRNA 43. Rather than directly binding m^6^A modification, HNRNPG recognizes the m^6^A methyl group that has remodeled its surrounding RNA sequence by m^6^A switch regulation, thus affecting the mRNA maturation 44.

**Figure 1:**
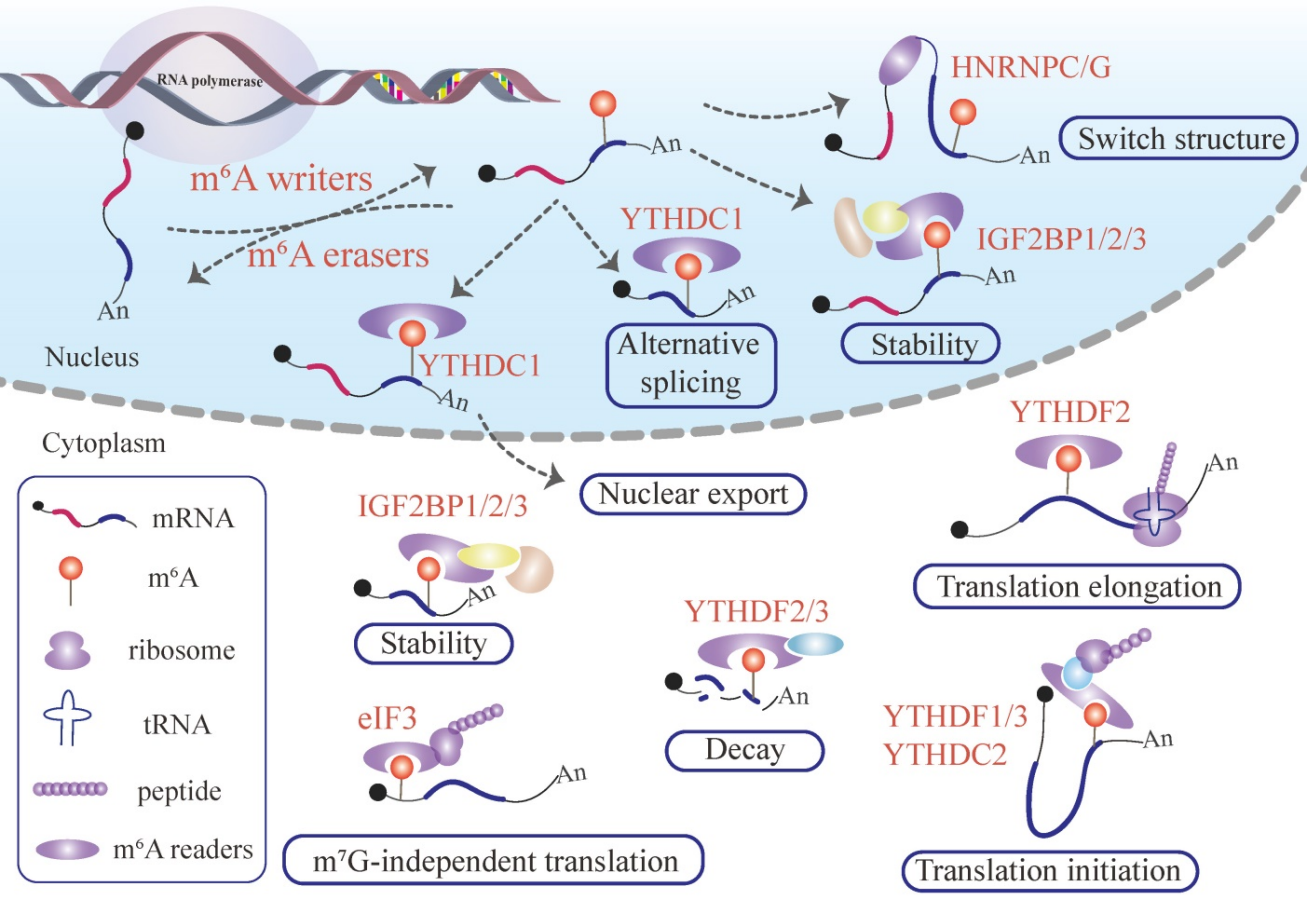
** Functions of m^6^A modification in mRNA.** Writer proteins deposit m^6^A remarks in newborn mRNAs transcript from DNA.The m^6^A signal deposited in mRNA can adjust the local flanking sequences by the m^6^A flag installed in the mRNA and recruit YTHDC1, which manipulates mRNA alternative splicing and nuclear export. The mRNA labeling m^6^A can recruit IGF2BPs to stabilize the mRNA in the nucleus. After penetrating the cytoplasm, the m^6^A-labeled mRNA produces sophisticated and far-reaching biological effects. IGF2BP1/2/3 proteins recognize the m^6^A remark embedded in mRNA, influencing the mRNA stability. On the contrary, m^6^A deposited in mRNA can recruit YTHDF2/3, promoting target mRNA degradation. In addition, the presence of an m^6^A modified mRNA regulates target mRNA translation and, consequently protein synthesis. The binding of eIF3 and m^6^A pockets launches m^7^G-independent translation. The interplay between YTHDF2 and m^6^A sites existing in mRNA enhances translation elongation. YTHDF1/3 or YTHDC2 perceive m^6^A pockets, and promotes target mRNA translation initiation.

Furthermore, the final fate of the m^6^A labeled mRNA depends on the location of m^6^A modification in mRNA transcripts and the type of m^6^A readers recognizing the m^6^A site (Figure 1). The identification of functional and novel m^6^A regulators as well as the deep deciphering of working mechanisms will require more advanced and mature high-throughput approaches for profiling RNA-protein interactions.

### 1.4 Emerging m^6^A -specific regulatory mechanisms

To date, most studies have mainly focused on the delicate and complex function of m^6^A proteins. However, the internal regulatory mechanisms of these writers, erasers and readers remain largely unknown. Exemplifying the upstream regulatory mechanism of these proteins will enable a better comprehension of the far-ranging biological effects of m6A and provide guidelines for m6A-associated diseases. A recent study has found the methyltransferase activity of METLL3 decreases following SUMOylation modification 45. However, other characteristics, including stability, localization, and interplay with METTL14 and WTAP do not undergo substantial alteration 45.

Further research has revealed that extensive SUMOylation occurring in the 177/211/212/215 lysine sites of METTL3 spatially affects its interaction with substrate mRNAs, which alternatively reduces methyltransferase activity 45. Subsequently, the authors have again validated that the SUMOylation accepted by the 571-lysine site of YTHDF2 enhances its binding affinity with m6A-bearing mRNA, which contributes to the decay-promoting effect of YTHDF2. In addition, this SUMOylation could be activated by microenvironmental hypoxia 46. Herein, we theorize that m6A regulators undergo further post-translational modifications (ubiquitination, phosphorylation, acetylation, and so on). These special modifications may play a direct role in the function of m^6^A modified RNAs under specific stress conditions or pathological conditions.

Surprisingly, site-specific m^6^A modifications mechanisms and their predilection to occur in the CDS and 3'UTR of mRNA have been recently uncovered. Research has found that H3K36me3 histone modification directly recognizes METTL14 (serves as an m^6^A reader), thereby co-transcriptionally governing m^6^A deposition 47. However, it is notable that almost all were actively transcribing genes with substantial H3K36me3 modifications, whereas m^6^A modification varies widely across mRNAs. How do they achieve their selectivity? The lack of a structural domain for METTL14 to recognize H3K36me3, indicates that a novel recognition mechanism achieves required to bind H3K36me3 to METTL14, and the structural biological basis of this interaction must to be deeply explored.

## 2. The role and regulation of m^6^A in ncRNAs

### 2.1 m^6^A in miRNA

**Figure 2:**
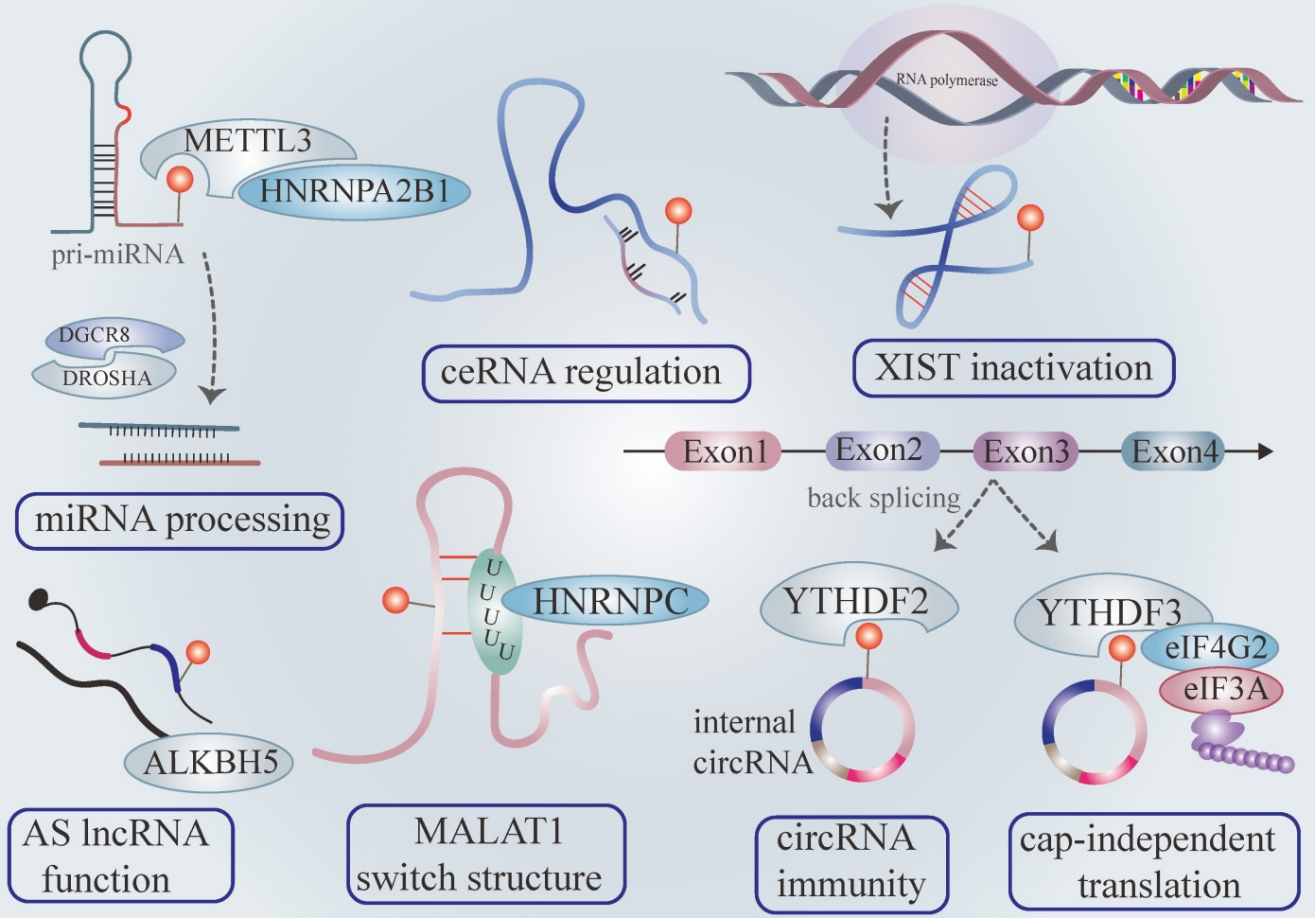
** The roles of m^6^A modification in non-coding RNAs.** METTL3 and hNRNPA2B1 promote pri-miRNA processing and mature miRNA output in the m^6^A tone. The m^6^A modification existing in long noncoding RNA influences its binding affinity with target miRNA, influencing the target mRNA of boundary miRNA (also called competitive endogenous RNA working mechanisms). YTHDC1 zooms lncRNA XIST -mediated gene silence. The m^6^A modification acts as a controlling element to regulate AS lncRNA functions. The m^6^A label in lncRNA also change the secondary structure via the m^6^A switch. The m^6^A signal existing in circRNA could be recognized as “self” circRNA, distinguishing itself from foreign circRNA and escaping RNA immunity. CircRNA launches cap-independent translation mediated by the YTHDF3/eIF4G2/eIF3A complex in an m^6^A dependent fashion.

miRNA accounts for most ncRNA and exert diverse functions in gene expression. miRNA biogenesis originates from the primary transcripts of miRNA transcribed from DNA (pri-miRNA) processing under the guideline of the microprocessor complex 48. The microprocessor complex subunit DGCR8 while ribonuclease type III DROSHA mediate primary mi-RNA processing 49. Specifically, DGCR8 recognizes the dsRNA-ssRNA junction of the pri-miRNA hairpin by its heme-bounding and RNA binding domains. It then recruits DROSHA, which releases hairpin-shaped pre-miRNA by cleaving 11 bp away from the junction 50. Furthermore, METTL3 endows pre-miRNAs with m^6^A modification and recruits DGCR8, facilitating pre-miRNA processing and mature miRNA output by triggering a global co-transcriptional procedure microprocessor machinery governing pri-miRNA processing 51 (Figure 2).

Consistent with the above findings, HNRNPA2B1 selectively recognizes the m^6^A motif of pri-miRNAs marked by METTL3 and interacts with DGCR8, enhancing pri-miRNA output via microprocessor machinery 25. The depletion of hNRNPA2B1 or METTL3 attenuates pri-miRNA processing 25 (Figure 2). Recent research has validated that METTL14 plays a manipulative role in pri-miRNA processing 52. It is widely accepted that miRNA directly binds 3'UTR of mRNA to exert biological function. Interestingly, the m^6^A peaks are strongly enriched in 3'UTR, implying a region-specific regulatory mechanism. Previous studies have revealed that m^6^A influences the binding affinity of miRNA with the 3'UTR mRNA 8. Furthermore, the miRNA or miRNA enzyme Dicer adversely regulates overall m^6^A level and individual m^6^A abundance of mRNA via interacting with the mRNA-METTL3 complex in an m^6^A-dependent pattern 53. The accumulation of mutation miRNA in mRNA binding sites conceives the novel m^6^A modification on the previously unmethylated sequence. Indeed, the augmentation of m^6^A production is indispensable for pluripotent cell programming 53.

### 2.2 m^6^A in lncRNA

Long non-coding RNA has been found to modulate gene expression by interacting with proteins, cross-talking with other RNA molecules, or remodeling chromatin despite lacking an open reading framework. The m^6^A mapping researchers have identified the chemical m^6^A modification present in the lncRNA sequence and confirmed that m^6^A manipulates the local structure and function 8, 41, 54. LncRNA X-inactive specific transcript (XIST) is well-known to mediate gene silence of the X chromosome at the transcriptional level by recruiting slicing proteins. RBM15/15B serves as a scaffold in anchoring the WTAP-METTL3 methylation complex to XIST, which catalyzes m^6^A modification in both adjacent and distant binding sites, depending on its three-dimension structure 55. The m^6^A mark of XIST can recruit YTHDC1, whose binding with XIST is indispensable for X-linked gene silence. Furthermore, METTL3 knockdown decreases m^6^A abundance and triggers X-linked gene silence 55. It is reasonable to assume that m^6^A modification is required for the proper XIST function (Figure 2). However, the molecular mechanism by which YTHDC1 binds to XIST to mediate a series of repressive chromosome programs remains unclear.

LncRNA binds to miRNA by RNA-RNA interaction, which relieves the suppression of miRNA on target mRNA, thereby alternatively enhancing mRNA functions 56. This mechanism is also the termed competitive endogenous mechanism (ceRNA). The m^6^A modification could fine-tune the ceRNA working mechanism (Figure 2). METTL3 endows linc1281 with an m^6^A signal and this m^6^A modification contributes to the binding of linc1281 with miR-let7, which indirectly increases target mRNA Lin28 abundance 57. As previously described, METTL3 can enhance the output of mature miRNA. It's important to distinguish the enhancement of binding affinity of linc1281 with miR-let7 is caused by METTL3 or m^6^A modification. The hairpin-stem structure of metastasized associated lung adenocarcinoma transcript (MALAT1) prefers to be m^6^A modified in 2577 residues, which destabilizes the opposing U5-tract structure within lncRNA MALAT1 41, 58. This enhances the accessibility of the U5-tract with HNRNP C, which intensifies the interactions between MALAT1 and HNRNP C via m^6^A-switch mechanism 41 (Figure 2). ALKBH5 up-regulates FOXM1 expression by removing its m^6^A modification and FOXM1-AS facilitates this regulation relationship 59. GATA3-AS, the lncRNA anti-sense to GATA3, contributes to the interaction between KIAA1429 and GATA3 nascent transcript 60. Here, we noted that interference with anti-sense lncRNA, transcribed from the antisense strand of the target mRNA, seems to function as a cis-acting element by regulating the preferential interaction between m^6^A proteins and pre-mRNA (Figure 2). However, how these anti-sense lncRNAs interact with m^6^A proteins remains elusive. Notably, over 70% of gene transcriptomes have been confirmed to produce anti-sense transcripts 61. Identifying the existence of other anti-sense lncRNA containing m^6^A modification will aid in understanding the distinct m^6^A level in certain genes.

### 2.3 m^6^A in circRNA

CircRNA is produced from a series of lariats back splicing events rather than the canonical linear splicing 62. Recently, overpowering evidence has shown that m^6^A modification abundance across different types of circRNA is determined by its exon feature 63. CircRNA consisting of long single exons contains more enriched m^6^A peaks than circRNA composed of multi-exons 63. The m^6^A-circRNA is created by mRNA exon lacking m^6^A, demonstrating that m^6^A-circRNA shares similar writers and readers (YTH proteins) with mRNA 16, 63. However, m^6^A-circRNA originated from a methylated exon that can recognize YTHDF2 inversely promotes parental mRNA degradation rather than circRNA 63. It appears that this regulatory mechanism guarantees a stable m^6^A-circRNA output. Research results have indicated that some circRNAs are intrinsically connected with polysomes, suggesting endogenous circRNA can be translated and carries coding potential 64, 65. Extensive m^6^A peaks in circRNA stimulate cap-independent translation by recruiting YTHDF3, eIF4G2, and eIF3A 66 (Figure 2). Interestingly, the authors discovered that this circRNA translation could significantly increase under heat shock conditions, where circRNA translation driven by m6A plays a key role in stress response 66. The exact function of protein or peptide generated by cap-independent m^6^A-circRNA translation needs to be further exemplified.

RNA immune system defines the internal and foreign RNA transcripts by checking the 5'm7G cap structure and RNA pattern recognition receptor I (RIG-I) necessary for internal immunity senses 5'-triphosphate in the ends 67. Large endogenous circRNA can escape this screening due to lacking ends. Indeed, the circRNA immune system can distinguish internal circRNA from foreign ones. Since internal circRNA was generated, it is marked by m6A 68. YTHDF2 binds to the m^6^A site to prevent removal of the m^6^A mark, thereby allowing the circRNA recognized to be as "self" (Figure 2).

In contrast, foreign circRNA is discriminated by RIG I and drives innate immunity owing to the lack of m^6^A modification 68. Interestingly, foreign circRNA can adjust itself to escape the RIG I screening by m^6^A modification, which makes foreign circRNA changed to be "self" 68. RNA chemical modifications besides m^6^A likely modulate circRNA immunity and further studies are warranted to elucidate their role.

## 3. Malfunctions of m^6^A of mRNA in cancer

**Figure 3:**
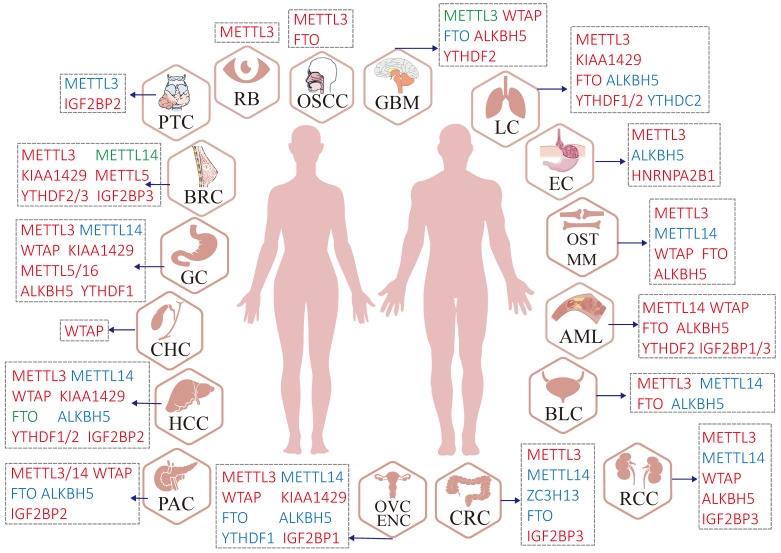
** Malfunctions of m^6^A regulators in various human cancers.** Red regulators suggest an oncogenic character. In contrast, blue regulators suggest a tumor-suppressive character and the roles of green regulators are hard to define (controversial portrayal reported in the specific cancer type).

It is widely accepted that m^6^A plays a key role in various biological processes such as neurogenesis development, directional differentiation of hemopoietic stem and progenitor cell, spermatogenesis, stress response, and RNA metabolism as previously described 23, 69, 70. Extensive efforts have been devoted to identifying the complex influence of m^6^A modification and its underlying regulatory mechanisms in multiple cancers (Figure 3). In addition, the deregulation of m^6^A proteins impacts multiple steps of tumorigenesis including initiation, metastasis, relapse, drug resistance, etc. 71, 72.

### 3.1 Malfunction of m^6^A writers in cancer

#### Oncogene

METTL3 has been reported to serve as an oncogene in most cancers, including HCC, CRC, AML, PAC, PRC, BRC, OVC, NSCLC, ESCC, etc. For instance, METTL3 facilitates NSCLC growth by increasing YAP translation through recruiting YTHDF1/3 and eIF3a. In addition, METTL3 enhances MALAT1 stability in an m^6^A manner, which promotes YAP expression by removing the suppression of miR-1914-3p 102. Accumulating evidence has demonstrated the oncogenic effect of METTL14 in AML, PAC, and NSCLC. METTL14 upregulation mediated by oncogenic protein SPI1 catalyzes m^6^A reaction on target mRNA MYB and MYC, attenuating HSPCs myeloid differentiation and enhancing self-renewal of leukemia stem/initiation cells (LSC/LICs), ultimately accelerating AML development 115.

WTAP has been identified to exhibit an oncogenic role in most tumors (Table 1). WTAP facilitates GC progression by binding with the 3'UTR m^6^A site of HK2 mRNA, which elongates HK2 transcript half-life to drive cell proliferation and glycolytic capacity 139. KIAA1429 is an oncogene in NSCLC, where KIAA1429 regulates m^6^A modification of death-associated protein kinase 3 (DAPK3) by YTHDF2/3-mediated degradation and enhances cell growth 148. Progressively, it has been reported that 18S RNA m^6^A methyltransferase METTL5 is highly expressed in BRC tissues and cells and promotes BRC development 151. Nevertheless, the underlying mechanisms remain an enigma. Emerging studies have documented that METTL16 is up-regulated in GC tissues and cells and related with a poor prognosis 153.

#### Tumor suppressor

As shown in Table 1, METTL14 and ZC3H13 have been reported to function as tumor suppressors in most cancer. Of note, METTL3 remodels neutrophil infiltration in the tumor microenvironment by regulating the m^6^A/c-Rel/IL-8 network, thereby inhibiting PTC progression 112. METTL14 is rarely expressed in BLC tissues and tumor-initiating cells (TIC) and knocking out METTL14 promotes cell survival, self-renewal, migration, and invasion by regulating Notch1 mRNA stability, which plays an important role in TIC-driven BLC tumorigenesis 116. METTL14 has been verified to be weakly enriched in GC tissues and a low METTL14 level predicts a poor prognosis 122. METTL14 slumps CRC progression by triggering m^6^A modification on oncogenic lncRNA XIST, and the m^6^A label in XIST is subsequently recognized by YTHDF2, which finally mediates XIST degradation 120. Likewise, ZC3H13 has been identified to suppress CRC tumorigenesis by regulating the Ras-Erk pathway 150. However, whether this tumor-inhibiting effect of ZC3H13 is dependent on its m^6^A modification or not remains unclear. Another study has revealed that METTL5 is downregulated in GC tissues and inhibits cancer development 152.

#### The dual effects of m^6^A writers on cancer

Notably, the exact role of METTL3 in GBM is intricate and difficult to define. In GBM, overexpressing METTL3 reduces CD44 expression and sphere-formation rate of glioblastoma stem cells (GSCs), indicating that it suppresses GSC growth and self-renewal, whose presence confers cell resistance to radiotherapy and chemotherapy 90. On the contrary, another study has reported that knocking down METTL3 promoted the SRSF-mediated nonsense-mediated mRNA decay effect, adversely altering BCL-X and NCOR2 splicing patterns, thereby leading to GSC apoptosis and differentiation 91. These discrepancies and controversies regarding METTL3 in GBM may depend on variable m^6^A reader protein and research contexts such as the origin of tumor sample, compensatory genetic mutation, and epigenetic influence of GSC cells in distinctive growth backgrounds. Similarly, the role of METTL14 in BRC is complex and hard to determine. METTL14 exhibits both oncogenic and tumor-suppressor effects in BRC according to different study data 11, 117, 118. Systematic and comprehensive BRC research is needed to clarify the actual influence of METTL14 in BRC.

There are currently a very limited number of investigations attempting to identify the influence of MTC adaptor proteins excluding WTAP and m^6^A methyltransferase in human cancers. A cross-sectional and comprehensive study is required to decipher the magic code of m^6^A modification in caner.

### 3.2 Malfunction of m^6^A erasers in cancer

#### Oncogene

FTO has been confirmed as an oncogene in cancers including AML, MM, OSCC, and NSCLC (Table 2). For example, FTO reduces the m^6^A level of E2F1 and increases E2F1 expression in NSCLC, which facilitates cell proliferation and metastasis by activating neural epidermal growth factor-like 2 (NELL2) transcription, and thus promoting tumorigenesis 162. ALKBH5 has been reported as an oncogene in AML, GC, OST, ENC, etc. (Table 2). Homeobox protein Hox-A10 (HOXA10) interacts with the TAAA region of ALKBH5 promoter as TF and expedites ALKBH5 expression, which adversely drives HOXA10 expression by mediating its stability 177. This ALKBH5-HOXA10 regulating loop promotes JAK2 demethylation, insulating the binding affinity of the m^6^A site in the JAK2 transcript with YTHDF2, which enhances JAK2 stability and upregulates OVC cell resistance to cisplatin through activating STAT3 phosphorylation reaction 177.

#### Tumor suppressor

To date, FTO has been confirmed to inhibit PAC, CRC, OVC, and GBM progression (Table 2). It prevents PAC initialization by restricting Wnt signal-mediated cell proliferation and metastasis through downregulating Praja ring finger ubiquitin ligase 2 (PJA2) mRNA decay 164. In CRC, FTO level is negatively associated with recurrence and prognosis 156. ALKBH5 acts as a tumor suppressor in PAC, BLC, EC, NSCLC, and HCC (Table 2). In PAC, ALKBH5 stymies tumor outgrowth and metastasis by stimulating PER1 transcription in an m^6^A-YTHDF2-mediated mRNA degradation style, which causes ATM phosphorylation and G2/M arrest by ATM/CHK2/p53/CDC25 signal axis 178. The p53 signal adversely intensifies ALKBH5 transcription, forming a feedback loop consisting of PER1-hampered p53 expression and p53-magnified ALKBH5 expression in regulating PAC cells malignancies 178.

#### The dual effect of m^6^A writers on cancer

The role of FTO in HCC is hard to define. One research team used long-term diethyl nitrosamine (DEN) to induce HCC in the hepatic FTO-deficient mice 159. Subsequently, they found that depletion of FTO amplifies HCC burden and that FTO exerts a protective function in HCC initiation by regulating m^6^A modification of CUL4A 159. In contrast, another study revealed that FTO is upregulated in HCC tissues and cells while silencing FTO prohibits cell growth and induces G0/G1 cycle arrest by remodeling PKM2 translation in HCC 160.

### 3.3 Malfunction of m^6^A readers in cancer

#### Oncogene

As presented in Table 3, most reader proteins seem to promote cancer progression. For instance, YTHDF1 is associated with HCC maintenance, where HIF-1α swells YTHDF1 transcription by mediating HIF-1α-binding site 1 (HBS1) and HBS3 site of HIF-1α interacting with the YTHDF1 promoter region under hypoxic conditions, which zooms autophagy-related tumor growth and metastasis 183. Compared with normal neural stem cell (NSC), GSC exhibits preferential YTHDF2 expression and dependency while YTHDF2 strikingly increases the stability of oncogene MYC and VEGFA transcripts by regulating m^6^A modification. This activates the IGFBP3 responding element to ameliorate GSC growth 189. Notably, in GSC, YTHDF2 maintains the target mRNA MYC and VEGFA stability, which is inconsistent with the findings of this study: the role of YTHDF2 in mRNA destabilization. There might be additional unknown regulatory factors working differently for various types of cells. We can inter that at least in certain cancer stem cells, YTHDF2 might indirectly stabilize some transcripts by destabilizing mRNAs encoding suppressors of other mRNAs. Research has also revealed that novel independent-cap translation mediated by m^6^A binding with 5'UTR of YTHDF3 lead to the strong enrichment of YTHDF3 in brain metastases in BRC 192. YTHDC2 is consistently deposited in radioresistant NPC cells and physically interacts with insulin-like growth factor 1 receptor mRNA (IGF1R), which subsequently activates downstream IGF1R-AKT/S6 signaling response elements urged by translation initiation of IGF1R in an m^6^A dependent tone 194.

HNRNPA2B1 depletion significantly prohibits cell proliferation by repressing cellular lipid increase mediated by fatty acid synthetic enzymes ACLY and ACC1 195. IGF2BP1 is highly enriched in high-grade serous ovarian cancer. It advances SRC activation through a protein-ligand- independent RNA form, which can expedite EMT by elevating ERK2 expression based on RNA-binding in OVC cells 198. In HCC, IGF2BP2 directly perceives the m^6^A pocket embedded in FEN1, which prolongs FEN1 mRNA stability and facilitates HCC progression 199. IGF2BP3 enhances DNA methylation-deregulated and RNA m^6^A reader-cooperating lncRNA (DMDRMR) stability, which reinforces the G1-S transition in the cell cycle 205. In addition, the interaction between DMDRMR and IGF2BP3 increases the stability of target genes such as extracellular matrix genes (COL6A1, LAMA5, and FN1) and CDK4 by m^6^A modification, thus promoting CRC development 205.

#### Tumor suppressor

YTHDC2 is marginally expressed in LUAD and associated with poor clinically prognosis 193. It executes a tumor-suppressive function in LUAD via enlarging solute carrier 7A11 (SLC7A11) degeneration. YTHDC2 blocks cystine uptake and perturbs the following antioxidant project, which curbs the sensitivity of the PDX mouse models to antioxidant administration 193.

## 4. m^6^A in the tumor microenvironment

Recently, the tumor environment (TME) characterized by hypoxia, aberrant metabolism, immune escape, and chronic inflammation has been reported to affect anti-tumor response. Accumulating evidence has demonstrated that metabolic dysfunction promotes the shift of immune cells from an anti-tumor state to a tumor-active state, facilitating cancer cells escaping from the monitoring system in TME. The intrinsic m^6^A modification has been confirmed to remodel TME by regulating the integral crosstalk and communications between various TME cellular components such as T cell, natural killer cell (NK), dendritic cell (DC), tumor-associated with macrophages (TAM), tumor-associated with fibroblast (TAF), and so on, affecting the anti-tumor immune response. This indicates m^6^A can be a novel immunotherapeutic target 206.

**Figure 4:**
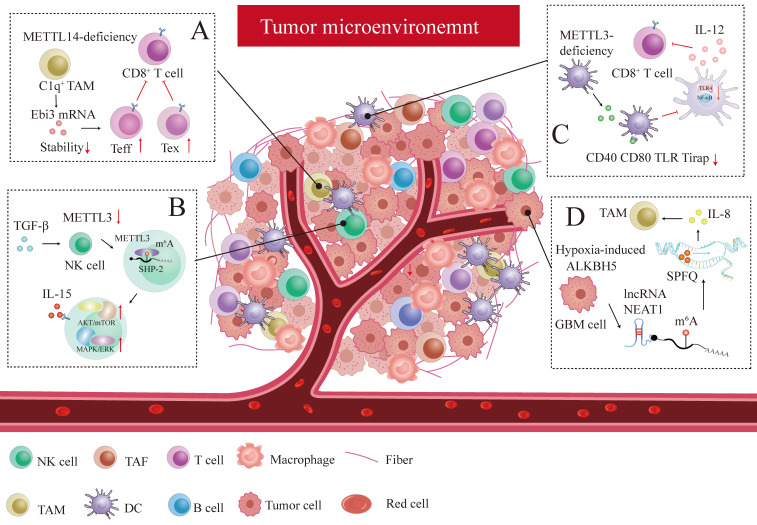
** The interplay between m^6^A modification and the tumor microenvironment (TME).** Several remarkable examples were reviewed below: (A) C1q^+^ TAM subpopulation-specific METTL14 deficiency induced tumor-infiltrating CD8^+^ T cell dysfunction by decreasing cytokine subunit Ebi3 mRNA stability, facilitating CD8^+^T cell exhaustion by increasing Tex cell and increasing Teff cell, and impeding CD8^+^T cells to eliminate tumors. (B) TGF-β inhibits METTL3 expression in tumor-infiltrating NK cells. By targeting SHP-2, METTL3 elevates the response of NK cells to IL-15 via the AKT-mTOR and MAPK-ERK signaling pathways, thus promoting the immunosurveillance of NK cells. (C) METTL3-deficiency in DC modulates co-stimulatory molecules (CD40 and CD80) and the translational efficiency of TLR signaling adaptor Tirap, which blocks T cell activation and responses by inhibiting cytokine IL-12 production mediated by TLR4/NF-κB signaling. (D) Hypoxia-induced GBM cell-specific ALKBH5 dramatically amplifies IL-8 production by eliminating m^6^A modification in lncRNA NEAT1, which triggers transcriptional suppressing factor SPFQ relocation from IL-8 promoter sequence to paraspeckles assembly mediated by NEAT1 stability.

Tumor-infiltrating T cell plays a pivotal role in the anti-tumor response. CD8^+^T cell subpopulations within the TME could be sorted into five discrete subpopulations: effector-like T cell (Teff), progenitor T cell, exhausted T cell (Tex), cycling exhausted T cell, and transitory T cell without enough activation according to their distinct signature gene profiles 207. C1q^+^ TAM subpopulation-specific METTL14 deficiency promotes tumor-infiltrating CD8^+^T cell dysfunction by reducing cytokine subunit Ebi3 mRNA stability, promoting CD8^+^T cell exhaustion by increasing Tex cell and increasing Teff cell count, dampening CD8^+^T cell to eliminate tumors 207 (Figure 4A). We can conclude that m^6^A plays a key role in deciding T cell fate within the TME.

METTL3 is downregulated in tumor-infiltrating NK cells within the TME caused by TGF-β, which activates m^6^A-mediated fragile effector function and limits the terminal differentiation of peripheral NK cells 208. METTL3 surges NK cells homeostasis and immunosurveillance by targeting SHP-2, which increases NK cell response to IL-15 via the AKT-mTOR and MAPK-ERK signaling pathways 208 (Figure 4B). Knocking out ALKBH5 in mouse model treated with GVAX/anti-PD-1 immunotherapy prohibited the nuclear export of metabolite content lactate, which latterly reduced the amounts of Treg cell and myeloid-derived suppressor cell (MDSC) and promotes DC infiltration by regulating mRNA MCT4/Slc16a3 expression in an m^6^A dependent fashion 209. YTHDF1 depletion enhances antitumor response and immunosurveillance by increasing the number of CD8^+^ cytotoxic T cells and infiltrating NK cells 210. DC-specific YTHDF1 enhances the neoantigen-specific immunity of CD8^+^T cells and inhibits the cross-presentation of engulfed tumor neoantigens by perceiving m^6^A pocket embedded in lysosomal proteases transcript, and enlarging translational output of lysosomal cathepsins 210.

Similarly, another research has uncovered that specific METTL3 absence in DC inhibits functional maturation and activation of DC by regulating co-stimulatory molecules CD40, CD80 and the translational efficiency of the TLR signaling adaptor Tirap, which curtails T cell activation and responses by suppressing cytokine IL-12 production mediated by TLR4/NF-κB signaling 211 (Figure 4C). Both studies have demonstrated that immune activation and tolerance induced by aberrant T cell response are closely associated with DC dysregulation. Adoptive infusion of DC vaccines could be particularly effective in cancer immunotherapy.

m^6^A affects immune checkpoint blockade (ICB) therapy and the efficacy of immunotherapy. Research has also revealed that the ALKBH5 expression pattern is consistent with the hypoxic phenotype signature-associated gene pattern in GBM cells 212. GBM cell-specific ALKBH5 induced by hypoxia stimulus significantly amplifies IL-8 production by clearing m^6^A modification in lncRNA NEAT1, provoking transcriptional suppressing factor SPFQ relocation from the IL-8 promoter sequence to paraspeckles assembly mediated by NEAT1 stability 212 (Figure 4D). The indirect IL-8-promoting effect mediated by hypoxic ALKBH5 expedites TAM recruitment within the TME and stymies the immunosuppressive microenvironment in anti-tumor response 212.

## 5. m^6^A regulator as a therapeutic target in cancer

**Figure 5:**
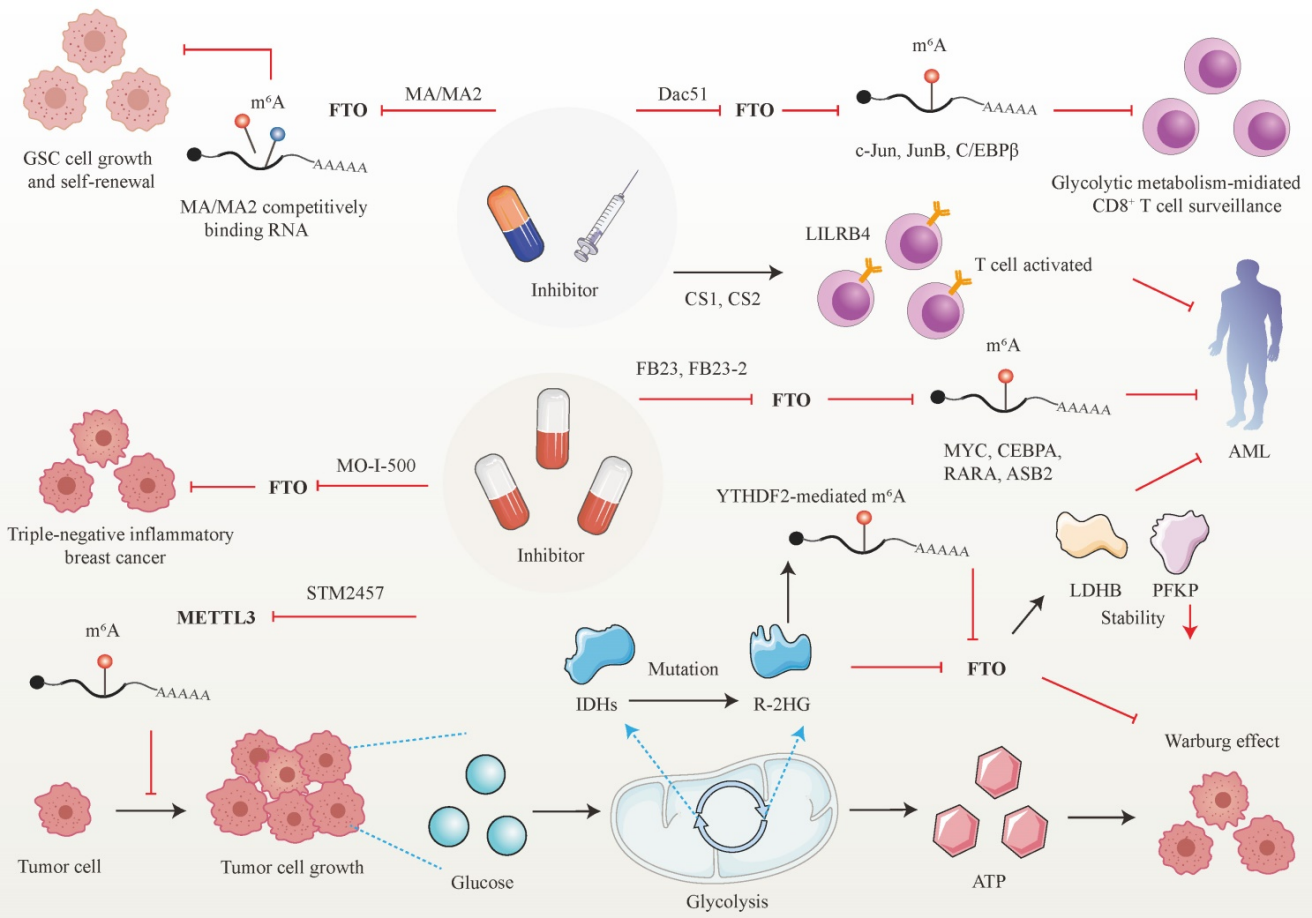
** Therapeutic implications of m^6^A regulators in cancer.** Given the attractive prospect of targeting FTO and METTL3 in cancer treatment, numerous inhibitors of m^6^A modifiers have been developed.

Developed small molecules targeting m^6^A writer and erasers, especially erasers in therapeutic applications have been designed and put into clinical practice considering the predominant role of m^6^A in cancer initiation and progression (Figure 5). Research groups have developed a bioavailable inhibitor of METTL3, namely STM2457, with highly potent and selective first-in-grade catalytic activity 213. Administration of STM2457 in AML re-transplantation experiments fazes tumor growth and facilitates survival by suppressing crucial stem cell flux and reversing AML phenotypes by inducing m^6^A-associated cellular effects including suppressing known leukemogenic mRNAs expression 213. According to a recent investigation, the small-molecule inhibitors FB23 and FB23-2 targeting FTO significantly suppress the proliferation of AML cell flux and primary LSCs in Xeon-transplanted mice by directly reducing m^6^A demethylase activity of FTO in target mRNA including MYC, CEBPA*, RARA* and* ASB2* 214. Two newly identified effective FTO inhibitors (CS1 and CS2) significantly repress the growth rate of LSC/LIC cells in vivo and in vitro by conquering the demethylation bag of FTO and hampering the handcuff of FTO in m^6^A-modified RNAs such as MYC 215. Notably, AML patients with high FTO expression phenotypes are highly responsive and sensitivite to C1 and C2 anti-leukemic treatments.

Interestingly, applying small-molecule compounds CS1/CS2 in AML cells catalyzes the cytotoxicity of the activated T cells to AML cells by regulating LILRB4, which serves as key intrinsic immune checkpoint genes to mediate immune evasion 215. Interestingly, C1 and C2 exhibit anti-tumor activity that is at least tenfold more potent than that of FB23 and FB23-2 in AML cells with IC_50_ values below nanomolar levels 215. Another small-molecule FTO inhibitor, Dac51, has also been confirmed to serve as an essential reprogramming RNA epitranscriptome factor to prevent tumor cells from evading CD8^+^T cells surveillance mediated by glycolytic metabolism, which is induced by transcription factors c-Jun, JunB, and C/EBPβ mediated by FTO in an m^6^A dependent tone 216.

Besides the above small-molecule inhibitors targeting m^6^A regulators, others targeting FTO have exhibited anti-tumor response in cancer therapy. For instance, R-2-hydroxyglutarate (R-2HG), a mutation of critical enzymes Isocitrate dehydrogenases (IDHs) in the aerobic glycolysis of cancer cells, has been reported to effectively blunt cancer metabolism by silencing the FTO-induced flux of transcription response associated with the Warburg effect 217. In R-2HG-sensitive leukemia cells, R-2HG suppresses FTO activity and decreases the stability of target mRNA phosphofructokinase platelet (PFKP) and lactate dehydrogenase B (LDHB) by YTHDF2. This minimizes aerobic glycolysis compared with healthy CD34^+^ hematopoietic progenitor cells, finally hampering AML metabolism and tumor growth 217. Consistent with the above findings, R-2HG also executes an anti-leukemic function by governing the FTO/m^6^A/MYC/CEBPA signal network. Moreover, R-2HG inhibits GBM tumor outgrowth and ameliorates tumor survival in vitro and in vivo by hindering FTO activity 218. The Saikosaponin D inhibitor targeting FTO has been indicated to decrease AML cell proliferation and tumor outgrowth, as well as resistance to tyrosine kinase inhibitors in vitro and in vivo by enhancing the overall m^6^A level of the downstream target genes 219. Pharmacological FTO inhibitor MO-I-500 suppresses cell proliferation and survival in triple-negative inflammatory breast cancer 220, 221. Meclofenamic acid (MA/MA2), a highly selective inhibitor of FTO, has been proved to faze GSC growth and self-renewal in vitro by competitively binding m^6^A-containing mRNA 90, 222.

## Conclusion and future perspectives

The latest proceedings in m^6^A epitranscriptome and technological advances in m^6^A associated methodology have conclusively validated that the dynamic and reversible m^6^A regulation network is indispensable for RNA metabolism including alternative splicing, nuclear export, stability, and translation. m^6^A emerges as a novel potential mechanisms of tumor-associated gene regulation. Remarkably, the dysregulation of m^6^A regulators in human cancer has been extensively documented. According to current reports, m6A writers, erasers, and readers are aberrantly expressed in most human cancer tissues and cells. These m^6^A regulatory factors manipulate malignancies such as unrestricted proliferation, active tissue metastasis and invasion, dysfunctional energy metabolism, skilled immune escape, and emerging resistance to chemotherapy and radiotherapy by governing oncogene or tumor-suppressor expression pattern in m^6^A dependent manner. However, how m^6^A writers and erasers distinctively perceive the onco-transcripts or tumor-suppressor transcripts and belatedly reshape most tumor-associated gene expression patterns remains an enigma. Fortunately, emerging studies have attempted to establish an explanation: the distinct cellular distribution of oncogenic or tumor-associated transcript and uneven dynamic m^6^A deposition, the cellular location of m^6^A readers and their variable amplification, cellular context and genetic background, and other additional unknown factors, these cross-talking elements orchestrate with each other to produce the m^6^A balance and exert diverse function. Most m^6^A proteins appear to facilitate tumor progression irrespective of the type of cancer. Intrinsically, extensive efforts have been made to elucidate the m^6^A-mRNA interaction. However, little is known about m^6^A modified ncRNA. Determining the interplay between m^6^A and ncRNA metabolism including ncRNA production, maturation, cellular location, and the cross-talks between m^6^A and ncRNA function in cancer cells will offer a profound understanding of the far-reaching influence of m^6^A and undervalued ncRNA. Fortunately, current report has highlighted the presence of m^6^A modification in promoter-related RNA, enhancer RNA, and other chromosome-associated RNA 31.

The tissue-specific m^6^A profiles of certain transcript and transcript loci could be promising biomarkers for the diagnosis and prognosis of cancer. More importantly, considering the critical and complex functions of m^6^A in cancer such as stemness, immune escape, energy metabolism, therapy resistance, and so on, endowing therapeutic target with m^6^A regulator goes one step further in clinical application. Current studies regarding m^6^A in cancer therapy seems extensively focused on AML and relevant studies performed on the other solid tumors are limited and remain largely unexploited. Moreover, the small molecules targeting m^6^A regulators including MO-I-500, MA, FB23-2, R-2HG, and CS1/CS2 are restricted to FTO. Therefore, potent and specific small molecule targeting m^6^A writers such as STM2457 based on small molecule inhibitor compound library screening should be of utmost relevance, considering that m6A writers mediate large m6A modified mRNAs in the whole genome. Furthermore, discoveries in cancer therapy targeting m^6^A readers controlling mRNA fate should not be ignored in the m^6^A research wave. Primary cell-derived from the target tissues, organoids, and animal models should be developed to test novel inhibitors' anti-tumor response, cellular toxicity, and pharmacokinetics. In the end, it is foreseeable that clinical trials of perfect small-molecule inhibitors targeting m^6^A regulators will be initiated and the pharmacological efficiency of mono/combination therapy in cancer patients with dysregulation of m^6^A profiles will be explored.

## Figures and Tables

**Table 1:** Roles of m^6^A writers in human cancer

	Cancer	Role	Expression	Target genes	Source of experimental evidence	Ref
**METTL3**	BLC	Oncogene	Up	PTEN, AFF4/NF-KB/MYC, SETD7/ KLF4, ITGA6	BLC tissues and cells, mouse	9, 73-75
	BRC	Oncogene	Up by HBXIP	miR-let-7, P21, miR-221-3p, KRT7	BRC tissues and cells	76-79
	CC	Oncogene	Up	HK2/YTHDF1, RAB2B, RAGE	CC tissues and cells, mouse	10, 80, 81
	CRC	Oncogene	Up	GLUT1/mTORC, miR-1246/SPRED2 /MAPK, IGFBP2, YPEL5, CCNE1	CRC tissues and cells, mouse	82-86
	ENC	Oncogene	Up	Akt	ENC tissues and cells, mouse	87
	ESCC	Oncogene	Up	Notch, APC	ESCC tissues and cells, mouse	88, 89
	*GBM*	Tumor suppressor/Oncogene	Down/Up	SRSF, ADAR1	GBM tissues and cells, mouse	90-92
	GC	Oncogene	Up by H3K27ac	HDGF/IGFBP2/GLUT4/ENO2, ZMYM1/Hur/CtBP/LSD1, MYC, miR-17-92/PTEN/TMEM127	GC tissues and cells, mouse	93-96
	HCC	Oncogene	Up	FOXO3, SOCS2, Snail, CRNNB1	HCC tissues and cells, mouse	97-99
	LC	Oncogene	Up	JUNB, miR-143-3p/VASH1	LC cells	100, 101
	NSCLC	Oncogene	Up	MALAT1/miR-1914-3p/YAP, ABHD11-AS1, ATG5/7	NSCLC tissues and cells, mouse	102-104
	OSCC	Oncogene	Up	c-MYC/YTHDF1, BMI1/IGF2BP1	OSCC tissues and cells, mouse	105, 106
	OST	Oncogene	Up	ATAD2	OST cell	107
	OVC	Oncogene	Up	miR-126-5p/PTEN/PI3K/Akt/mTor, miR-1246/CCNG2	OVC tissues and cells, mouse	108, 109
	PRC	Oncogene	Up	Akt, ITGB1, USP4	PRC cells, mouse	110, 111
	PTC	Tumor suppressor	Down	c-Rel/IL-8	PTC tissues and cells, mouse	112
	RB	Oncogene	Up	PI3K/Akt/mTOR	RB tissues and cells, mouse	113
	RCC	Oncogene	Up	ABCD1	RCC tissues and cells, mouse	114
**METTL14**	AML	Oncogene	Up by SPI 1	MYB/MYC	AML cells, mouse	115
	BLC	Tumor suppressor	Down	Notch1	BLC tissues and cells,	116
	*BRC*	Oncogene/Tumor suppressor	Up/down	CXCR4/CTP1B1,	BRC tissues and cells, mouse	11, 117, 118
	CC	Tumor suppressor	Down	Akt	CC cells, mouse	87
	CRC	Tumor suppressor	Down	miR-375, lncRNA XIST, SOX4	CRC tissues and cells, mouse	119-121
	GC	Tumor suppressor	Down	PI3K/AKT/mTOR	GC tissues and cells, mouse	122, 123
	HCC	Tumor suppressor	Down	DGCR8/miR-126, EGFR/PI3K/AKT	HCC tissues and cells, mouse	52, 124, 125
	NSCLC	Oncogene	Up	Twist	NSCLC tissues and cells	126
	OST	Tumor suppressor	Down	cas3	OST tissues and cells	127
	PAC	Oncogene	Up	PERP, mTOR	PAC tissues and cells, mouse	128, 129
	RCC	Tumor suppressor	Down	BPTF, lncRNA NEAT1	RCC tissues and cells, mouse	130, 131
**WTAP**	AML	Oncogene	Up	Hsp90	AML cells and mouse	132, 133
	CHC	Oncogene	Up	MMP7/MMP28	CHC tissues and cells, mouse	134
	ENC	Oncogene	Up	CAV-1/NF-κB	ENC tissues and cells	135
	GBM	Oncogene	Up	EGFR	GBM tissues and cells, mouse	136, 137
	GC	Oncogene	Up	HK2	GC tissues and cells, mouse	138, 139
	HCC	Oncogene	Up	ETS1/p21/27	HCC tissues and cells, mouse	140, 141
	OST	Oncogene	Up	HMBOX1/ PI3K/AKT	OST tissues and cells, mouse	142
	PAC	Oncogene	Up	Fak	PAC tissues and cells, mouse	143
	RCC	Oncogene	Up	CDK2	RCC tissues and cells, mouse	144
**KIAA1429**	BRC	Oncogene	Up	CDK1	BRC tissues and cells,mouse	145
	GC	Oncogene	Up	c-Jun	GC tissues and cells, mouse	146
	GCT	Oncogene	Up	DNA repair	GCT tissues and cells, mouse	147
	HCC	Oncogene	Up	GATA3/HuR	HCC tissues and cells, mouse	60
	NSCLC	Oncogene	Up	DAPK3	NSCLC tissues and cells,mouse	148
	PRC	Oncogene	Up	Lnc CCAT1 and CCAT2	PRC tissues and cells	149
**ZC3H13**	CRC	Tumor suppressor	Down	Ras-ERK	CRC tissues and cells	150
**METTL5**	BRC	Oncogene	Up		BRC tissues and cells	151
	GC	Tumor suppressor	Down		GC tissues	152
**METTL16**	GC	Oncogene	Up	CDK1	GC tissues and celle,mouse	153

**Table 2:** Roles of m^6^A erasers in human cancer

	Cancer	Role	Expression	Target genes	Source of experimental evidence	Ref
**FTO**	AML	Oncogene	Up	ASB2/RARA	AML cells and mouse	154
	BLC	Oncogene	Up	MALAT/miR-384/MAL2	BLC tissues and cells, mouse	155
	CRC	Tumor suppressor	Down	MTA1	CRC tissues and cells, mouse	156, 157
	GBM	Tumor suppressor	Down	FOXO3a	GBM tissues and cells, mouse	158
	*HCC*	Tumor suppressor/ Oncogene	Down/Up	PKM2	HCC tissues and cells.mouse	159, 160
	MM	Oncogene	Up	PD-1, CXCR4, SOX10	MM tissues and cells, mouse	161
	NSCLC	Oncogene	Up	E2F1/NELL2	NSCLC tissues and cells, mouse	162
	OSCC	Oncogene	Up	eIF4G1	OSCC tissues and cells, mouse	163
	OVC	Tumor suppressor	Down	cAMP	OVC tissues and cells, mouse	
	PAC	Tumor suppressor	Down	PJA2, Wnt	PAC tissues and cells,	164
**ALKBH5**	AML	Oncogene	Up	TACC3	AML cells	165
	BLC	Tumor suppressor	Down	CK2α	BLC tissues and cells, mouse	166
	EC	Tumor suppressor	Down	pri-miR-194-2/ RAI1	EC tissues and cells, mouse	167
	ENC	Oncogene	Up	IGF1R	ENC tissues and cells	168
	GBM	Oncogene	Up	HR	GBM cells	169
	GC	Oncogene	Up	NEAT1	GC tissues and cells,	170
	HCC	Tumor suppressor	Down	LYPD1	HCC tissues and cells, mouse	171
	NSCLC	Tumor suppressor	Down	miR-107/LATS2/YAP	NSCLC tissues and cells, mouse	172
	OST	Oncogene	Up	PVT1, pre-miR-181b-1/YAP	OST tissues and cells, mouse	173, 174
	OVC	Oncogene	Up	miR-7/BCL-2, NF-κB, HOXA10 / JAK2	OVC tissues and cells, mouse	175-177
	PAC	Tumor suppressor	Down	PER1, WIF-1/ Wnt signaling, KCNK15-AS1	PAC tissues and cells, mouse	178-180
	RCC	Oncogene	Up	AURKB	RCC tissues and cells, mouse	181

**Table 3:** Roles of m^6^A readers in human cancer

Cancer		Role	Expression	Target genes	Source of experimental evidence	Ref
**YTHDF1**	GC	Oncogene	Up	FZD7	GC tissues and cells and mouse	182
	HCC	Oncogene	Up	ATG2A, ATG14, FZD5	HCC tissues and cells and mouse	183, 184
	NSCLC	Oncogene	Up	CDK2, CDK4	NSCLC tissues and cells and mouse	185
	OVC	Oncogene	Up	EIF3C	OVC tissues and cells and mouse	186
**YTHDF2**	AML	Oncogene	Up	Tnfrsf2	AML cells, mouse	187
	BRC	Oncogene	Up	MYC	BRC cells,	188
	GBM	Oncogene	Up	MYC, LXRα/HIVEP2	GBM tissues and cells, mouse	189, 190
	HCC	Oncogene	Up	OCT4	HCC tissues and cells, mouse	12
	LUAD	Oncogene	Up	AXIN1/Wnt/β-catenin	LUAD tissues and cells, mouse	191
	PRC	Oncogene	Up	Akt	PRC tissues and cells, mouse	110
**YTHDF3**	BRC	Oncogene	Up	ST6GALNAC5/GJA1/EGFR	BRC tissues and cells, mouse	192
**YTHDC2**	LC	Tumor suppressor	Down	SLC7A11	LC tissues and cells, mouse	193
	NPC	Oncogene	Up	IGF1R/AKT/S6	NPC cells	194
**HNRNPA2B1**	EC	Oncogene	Up	ACLY, ACC1	EC tissues and cells	195
**IGF2BP1**	ENC	Oncogene	Up	PEG10	ENC tissues and cells, mouse	196
	AML	Oncogene	Up	HOXB4, MYB, ALDH1A1	AML cells, mouse	197
	OVC	Oncogene	Up	SRC/MAPK	OVC tissues and cells, mouse	198
**IGF2BP2**	HCC	Oncogene	Up	FEN1	HCC tissues and cells, mouse	199
	PAC	Oncogene	Up	PI3K/Akt	HCC tissues and cells, mouse	200
	PTC	Oncogene	Up	ERBB2	PTC cells,	201
**IGF2BP3**	AML	Oncogene	Up	MYC, CDK6	AML cells, mouse	202
	BRC	Oncogene	Up	miR-3614/ TRIM25	BRC tissues and cells, mouse	203
	CRC	Oncogene	Up	CCND1, VEGF	CRC tissues and cells, mouse	204
	RCC	Oncogene	Up	DMDRMR/CDK4	CRC tissues and cells, mouse	205

## Abbreviations

ncRNA: noncoding RNA
UTR: untranslated region
METTL3: methyltransferase-like3
METTL14: methyltransferase-like 14
MTC: methyltransferase complex
WTAP: Wilms tumor 1-associated protein
KIAA1429: via-like m^6^A methyltransferase associated
ZC3H13: zinc finger CCCH domain-containing protein 13
RAB15/15B: RNA-binding motif protein 15
METTL5: methyltransferase like 5
METTL16: methyltransferases like16
ZCCHC4: zinc finger CCHC-type containing 4
FTO: fat mass and obesity-associated protein
ALKBH5: ALKB homolog 5
AS: alternative splicing
mRNP: messenger ribonucleoprotein
HNRNPA2B1: heterogeneous nuclear ribonucleoproteins A2/B1
YTHDC1: YTH domain-containing protein 1
YTHDF1-3: YTH domain family proteins
IGF2BP1: insulin-like growth factor 2 mRNA-binding protein1
RBM: RNA binding motifs
XIST: X-inactive specific transcript
MALAT1: metastasized associated lung adenocarcinoma transcript 1
AML: acute myeloid leukemia
BLC: bladder cancer
BRC: breast cancer
CC: cervical cancer
CHC: cholangiocarcinoma
CRC: colorectal cancer
ENC: endometrial cancer
ESCC: esophageal squamous cell carcinoma
GBM: glioblastoma
GC: gastric cancer
GCT: germ cell tumors
HCC: hepatocellular carcinoma
LC: lung cancer
NSCLC: non-small cell lung cancer
OSCC: oral squamous cell carcinoma
OST: osteosarcoma
OVC: ovarian cancer
PAC: pancreatic cancer
PRC: prostatic cancer
PTC: papillary thyroid carcinoma
RB: retinoblastoma
RCC: renal cell carcinoma
LSC/LICs: leukemia stem/initiation cells
GSCs: glioblastoma stem cells
NPC: nasopharyngeal carcinoma
TME: tumor environment
NK cell: natural killer cell
DC: dendritic cell
TAM: tumor-associated with macrophages
TAF: tumor-associated with fibroblast
R-2HG: R-2-hydroxyglutarate
MA/MA2: meclofenamic acid
